# The potential of epigallocatechin gallate in the chemoprevention and therapy of hepatocellular carcinoma

**DOI:** 10.3389/fphar.2023.1201085

**Published:** 2023-05-24

**Authors:** Dongming Li, Donghui Cao, Yingnan Cui, Yuanlin Sun, Jing Jiang, Xueyuan Cao

**Affiliations:** ^1^ Department of Gastric and Colorectal Surgery, General Surgery Center, The First Hospital of Jilin University, Changchun, Jilin, China; ^2^ Division of Clinical Epidemiology, The First Hospital of Jilin University, Changchun, China

**Keywords:** epigallocatechin gallate, hepatocellular carcinoma, cancer prevention, cancer therapy, anticancer

## Abstract

Hepatocellular carcinoma (HCC), one of the most notorious malignancies globally, has a high fatality and poor prognosis. Though remarkable breakthroughs have been made in the therapeutic strategies recently, the overall survival of HCC remains unsatisfactory. Consequently, the therapy of HCC remains a great challenge. Epigallocatechin gallate (EGCG), a natural polyphenol extracted from the leaves of the tea bush, has been extensively investigated for its antitumor effects. In this review, we summarize the previous literature to elucidate the roles of EGCG in the chemoprophylaxis and therapy of HCC. Accumulating evidence has confirmed EGCG prevents and inhibits the hepatic tumorigenesis and progression through multiple biological mechanisms, mainly involving hepatitis virus infection, oxidative stress, proliferation, invasion, migration, angiogenesis, apoptosis, autophagy, and tumor metabolism. Furthermore, EGCG enhances the efficacy and sensitivity of chemotherapy, radiotherapy, and targeted therapy in HCC. In conclusion, preclinical studies have confirmed the potential of EGCG for chemoprevention and therapy of HCC under multifarious experimental models and conditions. Nevertheless, there is an urgent need to explore the safety and efficacy of EGCG in the clinical practice of HCC.

## Introduction

Liver cancer, particularly hepatocellular carcinoma (HCC), is one of the most frequent malignant tumors worldwide and a major threat to human health. Statistics shown that in 2020 alone, nearly 1 million patients were diagnosed with HCC globally (Sung et al., 2021). Currently, HCC is recognized as one of the top five principal causes of cancer-related death worldwide with relative consistency between morbidity and mortality annually, and the 5-year survival for most patients is less than 20% (Chidambaranathan-Reghupaty et al., 2021; Siegel et al., 2022). These statistical data suggest the treatment and prognosis of HCC remain extremely unsatisfactory. Chronic viral hepatitis B and C, cirrhosis, alcohol, fatty liver disease, diabetes, and aflatoxins are major hepatocarcinogenic risk factors that should be focused on prevention (Yang J. D. et al., 2019). Furthermore, several drugs have been confirmed to have some chemoprevention against HCC, such as aspirin, metformin, pioglitazone, statin, and obeticholic acid, but they seem to lack evidence of clinical benefit, instead, multiple side effects (Lange et al., 2021; Goh and Sinn, 2022; Zeng et al., 2023). Up to now, surgical resection, local ablation, liver transplantation, transcatheter interventional treatment, and systemic treatment are still the main therapeutic strategies for HCC with different clinical stages (Forner et al., 2018). Although recent breakthroughs have been made in systemic therapy of HCC, such as the establishment of standardized protocols based on immune checkpoint inhibitors (ICIs), most patients may lack a response to therapy and ultimately succumb to this cancer (Vogel et al., 2022). Consequently, the therapy of HCC remains a huge challenge for global healthcare, which suggests the demand to explore novel antitumor drugs.

Recently, the anti-cancer effects of natural compounds extracted from plants, especially tea trees, have been the fundamental area of concern to researchers. Globally, tea has become one of the oldest and most prevalent beverages. There are various kinds of tea, mainly divided into green tea, black tea, and oolong tea (Abudureheman et al., 2022). Abundant studies have unveiled the natural compounds in green tea have significantly antioxidant properties that are beneficial to health and reduce the risk of multiple diseases, such as inflammation, infection, cardiovascular disease, and cancer (Kochman et al., 2020; Musial et al., 2020). The pivotal reason for this is that green tea is made from the fresh leaves or buds of tea trees via fixation, twisting, and drying processes without the fermentation of black tea and oolong tea, thus retaining many nutrients of the tea, such as catechins, methylxanthines, amino acids and vitamins, among which catechins content is the most abundant, accounting for up to 30% of the dry weight of green tea (Kim et al., 2020; Ferrari et al., 2022; Trisha et al., 2022). On the basis of the different molecular structure, catechins are categorized into epigallocatechin gallate (EGCG), epigallocatechin (EGC), epicatechin gallate (ECG), gallocatechin gallate (GCG), epicatechin (EC), gallocatechin (GC), catechin gallate (CG), and catechin (C) (Li et al., 2018; Zhang et al., 2019b; Ferrari et al., 2022). Among these compounds, EGCG is the most bioactive and abundant, accounting for 50%–80% of all catechins in green tea (Samavat et al., 2019; Almatroodi et al., 2020; Ferrari et al., 2022).

EGCG, a polyphenol compound synthesized by esterification of epigallocatechin and gallic acid, consists of a dihydropyran heterocycle ring and 3 aromatic rings, where a total of 8 phenolic hydroxyl groups are contained (Bimonte et al., 2019). Since the polyphenol structure of EGCG allows electron delocalization to quench free radicals, EGCG is endowed with strong antioxidant capacity (Alam et al., 2022). Evidence has demonstrated that EGCG not only interacts with a variety of proteins, but enters the nucleus to regulate gene expression (Li et al., 2018). Based on these premises, numerous researchers have investigated the antitumor properties of EGCG recently. An expanding body of studies has revealed the potential of EGCG in chemoprophylaxis and therapy of multiple malignancies (Huang Y. J. et al., 2020; Romano and Martel, 2021; Wang et al., 2023).

In this article, we reviewed the potential role of EGCG in HCC. We comprehensively introduce the roles of EGCG in the chemoprevention and therapy of HCC as well as its molecular mechanisms *in vivo* and *in vitro* from multiple perspectives, such as hepatitis virus infection, hepatic tumorigenesis, oxidative stress, proliferation, invasion, migration, angiogenesis, apoptosis, autophagy, and metabolism, in order to provide new contributions to its clinical application.

## The roles of EGCG in chemoprevention and anti-cancer *in vitro*

**FIGURE 1:**
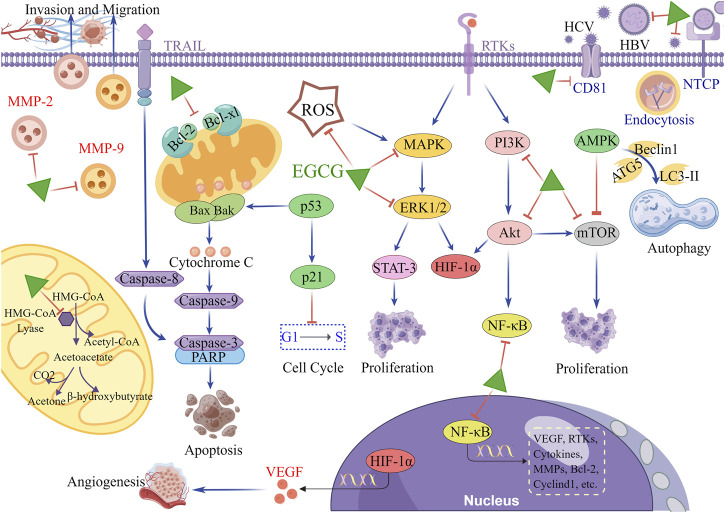
The main molecular mechanism of EGCG in HCC. When hepatocytes are exposed to hepatitis virus, EGCG inhibits the viral entry into the cells by reducing the viral adherence and downregulating viral receptors on the surface of hepatocytes. In terms of antitumor, EGCG downregulates multiple tumor-related molecules and signaling pathways, including RTKs, MAPK/ERK, PI3K/Akt, mTOR, NF-κB, STAT-3, HIF-1α and MMPs, to inhibit proliferation, angiogenesis, invasion, and migration. Simultaneously, EGCG upregulates p21 expression to induce cell cycle arrest. Additionally, the antitumor effects of EGCG are manifested in the stimulation of apoptosis through activating mitochondrial cytochrome C and TRAIL/TRAILR pathways. The activation of AMPK pathway is also involved in the regulation of EGCG in autophagy of hepatoma cells. In mitochondria, EGCG blocks the metabolic reprogramming of hepatoma cells by inhibiting the activity of HMG-CoA lyase, thereby reducing the production of ketone body. This figure was drew using Figdraw.

Accumulating preclinical evidence has suggested the chemoprophylactic and anti-cancer properties of EGCG involve multiple molecular targets and tumor-related signaling pathways (Figure 1). We discussed the antiviral and antitumor effects of EGCG *in vitro*, and devoted ourselves to clarifying its mechanisms in regulating proliferation, invasion, metastasis, angiogenesis, apoptosis, autophagy, and metabolism of hepatoma cells (Table 1).

**TABLE 1:** The main roles of EGCG in chemoprevention and anti-cancer *in vitro.*

Cell line	Main findings	Dose of EGCG	Main targets and signaling pathways involved	References
HuS-E/2	HBV Infection ↓	5, 10, 20 μM	None	Lai et al. (2018)
HepG2.2.15	HBV Infection ↓	12.5, 25, 50 μM	ERK1/2; HNF4α ↓	Wang et al. (2020)
HepG2.2.15	HBV Infection ↓	25, 50, 100, 200, 400, 800 μg/ml	None	Pang et al. (2014)
HepG2, HEK-293T	HBV Infection ↓	0–100 μM	FXRα	Xu et al. (2016)
HuS-E/2, HepG2.2.15	HBV Infection ↓	0–200 μM	NTCP	Huang et al. (2014)
HepG2, HepG2.2.15	HBV Infection ↓; Autophagy ↑	12.5, 25, 50, 100, 200 μM	LC3-I, LC3-II ↑; p62 ↓	Zhong et al. (2015)
Huh7	HCV Infection ↓	1, 5, 10 μM	IFN-λ1, TLR3, RIG-I, ISGs ↑	Wang et al. (2016)
Huh7	HCV Infection ↓	1, 5, 10 μM	IFN-λ1, TLR3, RIG-I, ISGs ↑	Wang et al. (2017b)
Huh-7, HEK-293T, Vero, MDBK	HCV Infection ↓	0–200 μM	Virus Particles	Calland et al. (2012)
Huh7	HCV Infection ↓	10 μg/ml	miR-548m ↑; CD81 ↓	Mekky et al. (2019)
Huh7	HCV Infection ↓	10 μg/ml	miR-194 ↑; CD81 ↓	Mekky et al. (2015)
None	Antiviral; Antioxidant	Unknown	HCV Protease ↓; ROS ↓	Zhong et al. (2012)
AH109A	Antioxidant; Invasion ↓	10, 50 μM	ROS ↓	Zhang et al. (2000)
HepG2, Hep3B	Antioxidant; Invasion ↓; Migration ↓	0–60 μM	ROS ↓; miR483-3p ↓; E-cadherin ↑; Vimentin ↓	Kang et al. (2021)
HepG2	Proliferation ↓	20, 40 μg/ml	IGF-1, IGF-2 ↓; IGF-1R ↓; ERK, Akt, STAT-3, GSK-3β ↓	Shimizu et al. (2008)
HepG2, SMMC-7721	Proliferation ↓	20 μM	IGF-1R, GRP78 ↓	Yin et al. (2017)
L-02, Hep3B	Proliferation ↓; Apoptosis ↑	0–100 μg/ml	ERα36, EGFR, HER2 ↓; MAPK/ERK ↓; PI3K/Akt ↓; Caspase-3 ↑	Chen et al. (2019)
Huh7	Proliferation ↓	0–100 μg/ml	VEGF, VEGFR-2 ↓	Shirakami et al. (2009)
HepG2	Proliferation ↓; Apoptosis ↑	0–100 μg/ml	VEGF ↓; Cyclin D1, Glypican-3 ↓; Caspase-3 ↑; Survivin ↓	Sabry et al. (2019)
HepG2	Proliferation ↓	10 μM	EGFR ↓; Cyclin D1, CYP2E1 ↓; NF-κB ↓	Shan et al. (2014)
Hep G2 (p53+), Hep 3B (p53-)	Proliferation ↓; Apoptosis ↑; Protein Synthesis ↓; Lipid Synthesis ↓	0–160 μM	AMPK ↑; mTOR ↓; p21 ↑; Cleaved PARP ↑; ACC, FASN ↓	Huang et al. (2009)
HepG2, HCT-116	Proliferation ↓; Apoptosis ↑	0–75 μM	CD133, NANOG ↓; Nek2 ↓; Akt ↓	Wubetu et al. (2016)
SK-Hep-1	Invasion ↓; Migration ↓	0.5, 1, 5, 10, 20 μg/ml	MMP-2, MMP-9 ↓	Lee et al. (2007)
SK-Hep-1	Invasion ↓; Migration ↓	0–500 μg/ml	MMP-2, MMP-9 ↓	Roomi et al. (2010)
HCCLM6	Invasion ↓; Migration ↓	5,10,20 μg/ml	MMP-2, MMP-9 ↓	Zhang et al. (2013)
HepG2, MHCC-97L, MHCC-97H, L02	Migration ↓; Proliferation ↓	12.5–100 μg/ml	PGE2 ↓; EP1 ↓	Jin et al. (2012)
HepG2	Migration ↓; Proliferation ↓; Apoptosis ↑	12.5, 25, 50, 100 μg/ml	EP1 ↓; Bax ↑; Bcl-2 ↓	Yang et al. (2019a)
HepG2, MHCC-97H, Hep3B	Migration ↓	0.02,0.2,2,20 μg/ml	Osteopontin	Zapf et al. (2015)
HepG2, Hela	Angiogenesis ↓	10,25,50,100 μM	HIF-1α↓; VEGF ↓; PI3K/Akt, ERK1/2 ↓	Zhang et al. (2006)
BEL-7402, QGY-7703	Proliferation ↓; Angiogenesis ↓; Apoptosis ↑	0–320 μM	STAT3 ↓; VEGF ↓; Bcl-xl ↓, c-Myc ↓	Wang et al. (2013)
HepG2, SMMC-7721, HUVEC	Angiogenesis ↓	0–200 μg/mL	HIF-1α↓; VEGF ↓; PI3K/Akt, MAPK/ERK ↓	Liao et al. (2020)
HUVEC, Huh7	Angiogenesis ↓	0–20 μM	VEGFR-2 ↓; MAPK/ERK ↓	Hashimoto et al. (2014)
HCCLM6	Apoptosis ↑	10, 30 μg/ml	Bcl-2 ↓; NF-κB ↓; p53↑; Bax, Caspase-9, Caspase-3 ↑	Zhang et al. (2015)
HepG2, SMMC-7721, SK-Hep1	Apoptosis ↑	74.7, 59.6, 61.3 μg/ml	PI3K/Akt ↓; NF-κB ↓	Shen et al. (2014)
HepG2	Apoptosis ↑	25, 50, 75, 100 μM	miR-16 ↑; Bcl-2 ↓	Tsang and Kwok (2010)
HLE, HepG2, HuH-7, PLC/PRF/5	Apoptosis ↑	10, 25, 50, 100 μg/ml	TRAIL; NF-κB ↓; Bcl-2, Bcl-xl ↓	Nishikawa et al. (2006)
HepG2	Apoptosis ↑	70 μg/ml	Caspase-3 ↑; Bcl-2↓; c-FLIP↓; DR4, DR5 ↑	Abou El Naga et al. (2013)
HCC-LM3, Huh-7, HepG2, Hep3B, SMMC-7721, LO2	Apoptosis ↑; Aerobic Glycolytic ↓	25, 50, 100, 200, 400 μM	PFK; Caspase-9, Caspase-8, Caspase-3 ↑; Bad, Bax ↑; Bcl-2, Bcl-xl ↓	Li et al. (2016)
HepG2	Autophagy ↑	0–150 μM	LC3-II ↑; LC3-I Dimer ↓; AFP ↓	Zhao et al. (2017)
HepG2, Huh7	Autophagy ↑; Lipid Clearance ↑	10, 20, 40 μM	LC3-II↑; AMPK ↑	Zhou et al. (2014)
Hep3B	Autophagy ↓	0–80 μg/ml	ATG5 ↓; Beclin1 ↓	Chen et al. (2014)
HepG2	Ketone Body ↓	0–100 μM	HMG-CoA lyase ↓	Nakagawa et al. (2013)

ISGs, IFN-stimulated genes; GSK-3β, glycogen synthase kinase-3β; CYP2E1, cytochrome P4502E1; PARP, poly (ADP-ribose) polymerase; ACC, acetyl CoA carboxylase; FASN, fatty acid synthase; c-FLIP, cellular FLICE-like inhibitory protein; DR4, TRAIL death receptor 4; DR5, TRAIL death receptor 5.

### Antiviral activities

Hepatitis B virus (HBV) is an extremely crucial pathogenic factor in cirrhosis and HCC (McGlynn et al., 2021). HBV, a hepatotropic DNA virus, not only causes chronic progressive hepatocyte damage, but integrates viral DNA into the host genome, thus inducing genetic alteration to result in HCC (Yang G. et al., 2022; Fung et al., 2022). According to reports, 10%–25% of HBV carriers undergo the hazard of developing HCC during their lifetime (McGlynn et al., 2015). However, globally, there is still a lack of highly satisfactory antiviral treatments. Evidence has revealed EGCG dose-dependently inhibits HBV-DNA replication and hepatitis B surface antigen (HBsAg) mRNA levels in immortalized human primary hepatocytes infected with HBV (Lai et al., 2018). Similarly, it has been demonstrated that EGCG can significantly block viral DNA replication and antigens expression in HepG2.2.15 cells through downregulating hepatocyte nuclear factor 4α (HNF4α) that are mediated by the extracellular signal-regulated kinase (ERK) signaling pathway (Wang et al., 2020). Even, the inhibitory effects of EGCG on the secretion of HBsAg, hepatitis B e antigen (HBeAg) may be stronger than lamivudine (Pang et al., 2014). As a vital transcriptional regulatory factor, EGCG can also effectively inhibit HBV promoter transcription through antagonizing farnesoid X receptor alpha (FXRα), a member of liver-enriched transcription factors that regulate transcription of liver virus mRNA, thereby reducing HBV replication (Xu et al., 2016). Notably, EGCG may effectively prevent HBV from entering hepatocytes by regulating endocytosis and degradation of sodium taurocholate co-transporting polypeptide (NTCP), the HBV receptors on the cell membrane, thus blocking a substantial proportion of HBV infection (Huang et al., 2014). Moreover, previous study has found EGCG can mediate lysosome acidification in hepatocytes, thus interfering with incomplete autophagosome formation necessary for HBV replication (Zhong et al., 2015).

In addition to HBV, hepatitis C virus (HCV) is another infamous pathogen causing HCC (Shen et al., 2023). In total, it is estimated that there are about 150 million patients who suffer from HCV infection worldwide (Luna-Cuadros et al., 2022). Studies have shown EGCG significantly enhances antiviral innate immune response in hepatocytes by inducing the expression of toll-like receptor 3 (TLR3), retinoic acid-inducible gene I (RIG-I), and interferon lambda 1 (IFN-λ1) (Wang et al., 2016; Wang Y. Z. et al., 2017). Additionally, EGCG can also effectively inhibit HCV entry into hepatocytes. Ample evidence has confirmed the potential mechanism by which EGCG retards HCV infection is that EGCG directly changes the viral particle structure of HCV, ultimately reducing the ability of HCV to adhere to the hepatocyte surface (Ciesek et al., 2011; Calland et al., 2012; Calland et al., 2015). HCV envelope proteins or capsid proteins may be involved in this process (Mathew et al., 2014; Shahid et al., 2021). Different from its direct effect on virus particles, EGCG may also inhibit CD81 receptors that is a critical pathway for HCV to enter hepatocytes through upregulating the expression of certain miRNAs, such as miR-548m and miR-194, thus reducing HCV infectivity in Huh7 cell lines (Mekky et al., 2015; Mekky et al., 2019). Furthermore, EGCG is far superior to HCV embelin, an effective HCV protease inhibitor, in inhibiting the activity of HCV NS3/4A protease that is the necessary enzyme to promote HCV shearing and maturation (Zhong et al., 2012).

Briefly, EGCG, as a valuable natural compound against liver virus, has the effective chemoprophylaxis against HCC. Hence, we consider that EGCG combined with antiviral drugs is promising as a novel therapy strategy for patients with viral hepatitis or liver cancer, especially for their prevention of viral reinfection after liver transplantation, but it remains to be further investigated.

### Antioxidative activities

The antioxidant capacity of EGCG depends on its polyphenol groups, which provide the prerequisite for electron delocalization, thus significantly quenching various reactive oxygen species (ROS) (Alam et al., 2022). This antioxidative activity is further elevated by the presence of the trihydroxyl structure in the aromatic ring of EGCG (Singh et al., 2011). As the main cause of cellular oxidative stress, ROS can damage DNA and cause accumulation of carcinogenic alterations, eventually initiating tumor pathogenesis (Srinivas et al., 2019). Concurrently, ROS plays the regulatory roles in tumor microenvironment (TME), cell metabolism, and immune response through a multiple signaling pathways, which are the crucial actions towards cell survival, proliferation, invasion, and metastasis in hepatoma cells (Cheung and Vousden, 2022). Evidence has suggested EGCG inhibits the adhesion and invasion based on its antioxidant capacity in ROS-potentiated AH109A cells induced by hypoxanthine and xanthine oxidase (Zhang et al., 2000). Besides, when the concentration of EGCG ranges from 10 μM to 30μM, EGCG can dose-dependently inhibit ROS generation induced by miR483-3p and attenuate the metastasis potential of HCC (Kang et al., 2021). Briefly, as a strong free radical scavenger, EGCG may inhibit hepatic tumorigenesis and progression by neutralizing intracellular ROS.

Interestingly, it is now realized EGCG also plays a prooxidant role under certain conditions, due to its chemical reactivity making it susceptible to generate ROS (Kang et al., 2008; Chen et al., 2020; Yang L. et al., 2022). Under cell culture conditions, when the medium containing EGCG is exposed directly to air, the phenol hydroxyl groups on the specific aromatic rings of EGCG are converted to o-quinone by autooxidation, yielding superoxide anion radicals and EGCG radicals, which may be beneficial for killing tumor cells (Ouyang et al., 2020). Besides, when the concentration of transition metal ions rises, EGCG can significantly chelate metal ions, including copper ions and iron ions (Bimonte et al., 2019). The chelation of free ions will hamper the antioxidant activity of phenol hydroxyl groups, resulting in EGCG autooxidation and hydrogen peroxide generation to induce cell apoptosis (Alam et al., 2022). Moreover, both EGCG concentration and pH in matrices determine which physicochemical properties EGCG should select (Zhou and Elias, 2013). In general, EGCG might represent an antioxidant at low doses (nanomolar levels), whereas EGCG manifested as a prooxidant at high doses (micromolar levels) in hepatic cells (Kang et al., 2008; Zhou and Elias, 2013; Ouyang et al., 2020). However, the specific critical concentration remains to be determined. Meanwhile, EGCG mainly acted as a prooxidant effect at low pH (pH ≤ 4), while its antioxidant effect was observed at relative high pH (pH = 7) (Zhou and Elias, 2012; 2013). Forementioned findings imply EGCG has dual effects on ROS regulation and may act as a prooxidant to play an antitumor cytotoxic role under certain conditions.

### Antiproliferative effects

The receptor tyrosine kinases (RTKs) are the pivotal factors in regulating physiological function of normal cell. Dysregulation of certain RTKs, especially insulin-like growth factor (IGF)-1 receptor (IGF-1R), epidermal growth factor receptor (EGFR), EGFR type 2 (HER2), and vascular endothelial growth factor (VEGF) receptor (VEGFR), are largely related to the acquisition of malignant hallmarks of tumor, as a result of which RTKs have become the most practical molecular targets for HCC therapy (Shimizu et al., 2011a; Park et al., 2022). Increasing evidence has proved EGCG can suppress the abnormal activation of multiple RTKs and their downstream signaling molecules in HCC. The possible mechanism is that it may bind directly or indirectly to RTKs, thereby blocking their tyrosine kinase activities. Specifically, EGCG reduces IGF expression and inhibits IGF-1R and corresponding downstream signaling molecules, such as ERK and phosphatidylinositol-3-kinase (PI3K)/protein kinase B (Akt), which are major signaling targets of RTKs activation, ultimately suppressing the proliferation of HCC through such IGF/IGF-1R axis (Shimizu et al., 2008; Shimizu et al., 2011a). Meanwhile, EGCG can directly inhibit downstream signaling of IGF-1R through acting on the adenosine triphosphate (ATP)-binding domain of 78 kDa glucose regulated protein (GRP78), a multifunctional chaperone protein that aggrandizes the phosphorylation and activation of IGF-1R (Yin et al., 2017). Recently, estrogen receptor alpha 36 (ERα36), a newly discovered estrogen receptor α-subtype in liver, which is prominently upregulated only in hepatoma cells but not in normal hepatocytes, has been recognized as a potential EGCG target in HCC. Evidence suggests EGCG can directly or indirectly target ERα36 in hepatoma cells, thus downregulating EGFR and HER2, as well as blocking PI3K/Akt and mitogen-activated protein kinases (MAPK)/ERK pathways (Chen et al., 2019). Moreover, VEGFR is significantly highly expressed in hepatoma cells, especially VEGFR-2, which is an essential regulator of tumor growth and angiogenesis (Bai et al., 2020; Teng et al., 2020). When researchers treated hepatoma cells with EGCG, VEGFR-2 expression markedly decreased in a time- and dose-dependent manner, implying EGCG may inhibit HCC cell growth by targeting VEGF-VEGFR axis (Shirakami et al., 2009). These findings suggest EGCG can hinder the proliferation of hepatoma cells by acting as an RTKs inhibitor.

Not only that, EGCG can also inhibit HCC growth by affecting cell cycle progression. EGCG significantly reduces the expression of cyclin D1 protein in hepatoma cells, which leads to cell cycle arrest, especially when combined with metformin (Shan et al., 2014; Sabry et al., 2019). Remarkably, the choice of EGCG between cell cycle arrest and apoptosis is largely dependent on p53 status. For p53-positive HepG2 cells, EGCG can induce G1-phase cell cycle arrest through increasing the expression of p53 and p21 successively, whereas EGCG can induce the apoptosis of p53-negative Hep3B cells, in which p21 is not affected by EGCG (Huang et al., 2009). Additionally, EGCG inhibits self-renewal of liver cancer stem cells (CSCs), weakens the stemness characteristics and disrupts the proliferation-related signaling molecules, including never in mitosis A (NIMA)-related kinase 2 (Nek2) and Akt in CSCs (Wubetu et al., 2016). Nevertheless, the regulatory mechanism of EGCG in CSCs remains obscure and needs to be further investigation.

### Antiinvasive and antimigratory effects

As the primary malignant hallmarks, invasion and metastasis bring severe challenges to HCC treatment. Several authors have confirmed matrix metalloproteinases (MMPs) participate in various processes of tumor progression, particularly tumor invasion and metastasis (de Almeida et al., 2022). MMPs remove barriers for invasion and migration by breaking the extracellular matrix and basement membrane (Huang et al., 2022). For instance, MMP-2 and MMP-9 can degrade type IV collagen in the basement membrane to help tumor cells invade blood vessels and lymphatic vessels (Cabral-Pacheco et al., 2020). Furthermore, MMPs induce the epithelial mesenchymal transformation of HCC cells to promote tumor invasion and migration (Scheau et al., 2019). Extensive evidence has confirmed EGCG suppresses HCC invasion and migration in a dose-dependent manner by reducing the activities of MMP-2 and MMP-9 (Lee et al., 2007; Roomi et al., 2010). Zhang et al. (2013) also has uncovered the inhibitory roles of EGCG in HCC metastasis relies on the suppression of MMP-2 and MMP-9 activities, but the detailed regulatory mechanism has not been explained yet. We speculate EGCG may directly bind to MMP-2 and MMP-9 to inhibit their activities, which has been supported by several additional studies (Sazuka et al., 1997; Jha et al., 2016; Saeki et al., 2018). It is worth noting that RNA-seq data analysis of hepatoma cells revealed EGCG downregulated the expression of some MMPs, such as MMP-11 and MMP-24 (Agioutantis et al., 2020). Given previous scientific research, we have also proposed some potential signaling pathways on how EGCG inhibits MMPs expression, including nuclear factor-kappaB (NF-κB), MAPK/ERK, and PI3K/Akt (Luo et al., 2017; Won et al., 2021; Tanabe et al., 2023).

EGCG also inhibits HCC invasion and migration through other pathways. Evidence has suggested prostaglandin E2 (PGE2) can bind to hepatocellular prostaglandin E receptors (EP1) to stimulate the progression and migration of hepatoma cells (Chen et al., 2022). EGCG can hinder the proliferation and migration of hepatoma cells though downregulating the expression of EP1 as well as the generation of PGE2 (Jin et al., 2012; Yang H. et al., 2019). Moreover, osteopontin, a turnover protein regulating extracellular matrix, is correlated with aggressiveness and prognosis of HCC (Song et al., 2021). Zapf et al. (2015) has revealed EGCG reduces the half-life of osteopontin mRNA, rather than affecting the transcription of osteopontin. This may be related to the attenuated migration of hepatoma cells. In brief, these results imply EGCG may inhibit malignant hallmarks of HCC by activating multiple molecular mechanisms.

### Antiangiogenic effects

Angiogenesis, a critical pathological hallmark in HCC, is the most practical therapeutic target for HCC (Morse et al., 2019). Evidence has suggested EGCG significantly reduces the expression of VEGF that directly acts on VEGFR on vascular endothelial cells and subsequently leads to the angiogenic dysregulation in HCC (Shirakami et al., 2009; Sabry et al., 2019). The relatively hypoxic environment inside the tumor can stimulate the expression of hypoxia-inducible factor-1 (HIF-1) to adapt to the hypoxic state in tumor tissue, thus activating various downstream target genes including VEGF (You et al., 2021). In hepatoma cells, EGCG inhibits hypoxia-induced HIF-1α accumulation through PI3K/Akt and ERK1/2 pathways, and subsequently causes a strong decrease in VEGF expression (Zhang et al., 2006). Additionally, EGCG directly inhibits signal transducer and activator of transcription 3 (STAT3), a complex transcriptional regulator that can activate HIF-1α expression and cooperate with HIF-1α in binding to the VEGF promoter to maximize the activation of transcription and angiogenesis (Wang et al., 2013; Tolomeo and Cascio, 2021). However, EGCG may have some limitations in anti-angiogenesis, such as relatively unstable chemical structure and poor bioavailability. To compensate for the deficiency of EGCG, EGCG derivative Y6 was synthesized through substituting ethoxy groups for six phenolic hydroxyl groups of EGCG (Zhou et al., 2020). Compared with EGCG, optimized EGCG more significantly downregulates HIF-1α and VEGF through inhibiting PI3K/Akt and MAPK/ERK1/2 pathways, thereby inhibiting angiogenesis of HCC (Liao et al., 2020; Zhou et al., 2020). Another derivative, methylated-EGCG, blocks VEGF-induced angiogenesis by significantly repressing the formation of tubular structures in human umbilical vascular endothelial cells, consistent with the validation in xenografted mice with liver cancer (Hashimoto et al., 2014). With the clarification of the abovementioned antiangiogenic mechanism, EGCG may become an auxiliary strategy for targeted therapy of HCC in future clinical practice.

### Proapoptotic effects

Abundant evidence has suggested EGCG-induced apoptosis is relevant to the apoptosis-related proteins. More narrowly, EGCG plays the prominent regulatory roles in a series of apoptosis-related signaling molecules in hepatoma cells, including upregulating the expression of p53, Bax, caspase-9, and caspase-3, downregulating the expression of NF-κB, survivin, and BCL2 apoptosis regulator (Bcl-2), and promoting the release of cytochrome c (Zhang et al., 2015; Yang H. et al., 2019; Sabry et al., 2019). Shen et al. (2014) has deciphered EGCG induces cell cycle arrest and apoptosis of hepatoma cells though suppressing PI3K/Akt pathway, in which the phosphorylation at Ser473 of Akt are decreased with EGCG treatment. Notably, some miRNAs can regulate the apoptotic induced by EGCG in hepatoma cells. For instance, miRNA microarray has unveiled EGCG enhances the levels of miR-16, which modulates EGCG-induced apoptosis by targeting Bcl-2 (Tsang and Kwok, 2010). Additionally, EGCG further induces apoptosis of hepatoma cell by coordinating tumor necrosis factor (TNF)-related apoptosis-inducing ligand (TRAIL) (Nishikawa et al., 2006). The reason may be that EGCG elevates the sensitivity of hepatoma cells to TRAIL-induced apoptosis by enhancing the expression of TRAIL receptor (Abou El Naga et al., 2013). Meanwhile, EGCG also directly inhibits the activity of phosphofructokinase (PFK), a glycolytic rate-limiting enzyme, resulting in metabolic stress-related apoptosis with the release of pro-apoptotic protein Bad (Li et al., 2016).

### Autophagy regulation

Previous investigation has found EGCG can induce autophagy of hepatoma cells by inhibiting the formation of microtubule-associated protein light chain 3 (LC3)-I dimer and promoting the synthesis of characteristic autophagic protein LC3-II, thus modulating the degradation of α-fetoprotein (AFP) that is relevant to malignant differentiation, metastasis, and poor prognosis of HCC (Zhao et al., 2017). Furthermore, EGCG induces autophagy in hepatoma cells by activating adenosine monophosphate-activated protein kinase (AMPK) pathway, which is mainly manifested as formation of autophagosomes, acidification of lysosomes, and enhancement of autophagic flux (Zhou et al., 2014). Nevertheless, Chen et al. elucidated EGCG could reduce the autophagy activity of Hep3B cells, which was mainly manifested as reduced autophagy vacuoles and downregulated expression of autophagic proteins, including autophagy-related protein 5 (ATG5) and beclin1 (Chen et al., 2014). This may be related to the reversely double effects of autophagy on tumors in distinct contexts and stages (Li et al., 2020). Overall, EGCG are likely to play an anticarcinogenic role through modulating autophagy of hepatoma cells.

### Metabolic regulation

To maintain the energy requirements of tumor cell growth and proliferation, tumor cells often initiate metabolic reprogramming to overcome the energy deficiency. As is known to all, ketone bodies are generated by the liver, but the liver of normal adults lacks the enzymes to consume them. However, studies have found that under nutritional deprivation of HCC, hepatoma cells can re-induce the synthesis of ketolytic enzymes to activate the ketone catabolism, which prevents excessive autophagy of hepatoma cells, thus promoting the progression of HCC (Huang D. et al., 2016). Simultaneously, β-hydroxybutyrate can induce the β-hydroxybutyrylation of p53, leading to an obvious decrease in the acetylation levels and activities of p53, which ultimately promote the growth of tumor cells (Liu et al., 2019). These evidence supports the regulatory roles of ketone bodies in HCC progression. EGCG can non-competitively block the activity of 3-hydroxy-3-methylglutaryl-coenzyme A (HMG-CoA) lyase that catalyzes the cleavage of HMG-CoA into ketone bodies in hepatoma cells (Nakagawa et al., 2013). This implies the anti-cancer effects of EGCG in HCC may involve the regulation of ketone metabolism. Furthermore, different from normal cells, tumor cells prefer aerobic glycolysis for energy despite the availability of oxygen, a phenomenon known as the “Warburg effect” (Zhou et al., 2021). EGCG suppresses the phosphofructokinase activity in hepatoma cells by converting its oligomer structure into an inactive state, thus resulting in aerobic glycolysis inhibition and tumor cell death (Li et al., 2016). Briefly, these findings suggest EGCG may inhibit the reprogramming of HCC energy metabolism. In addition to regulating energy metabolism, EGCG can inhibit the mechanistic target of rapamycin (mTOR) signal and the activities of lipogenic enzymes of hepatoma cells by activating AMPK pathway, subsequently obstructing the protein and lipid synthesis in HCC (Huang et al., 2009). EGCG can also induce autophagy stimulated by AMPK pathway to clear lipids, which promotes lipid metabolism in hepatoma cells (Zhou et al., 2014). Overall, EGCG affects the material and energy metabolism of HCC to a large extent.

## The roles of EGCG in chemoprevention and anti-cancer *in vivo*

*In vivo* animal experiments are the important basis to investigate the potential of EGCG in the prevention and therapy of HCC as they provide references to the pharmacological effects, which can subsequently be extrapolated to humans (Romano and Martel, 2021). We discuss the preventive and anti-cancer roles of EGCG *in vivo* from following perspectives (Table 2).

**TABLE 2:** The main roles of EGCG in chemoprevention and anti-cancer *in vivo.*

Experimental models	Method of administration	Dose of drug	Main effects	References
Female Hu-FRG mice	Tail vein injection	EGCG (50 mg/kg) was given twice a day for 5 consecutive days	Decreasing HBV DNA copy number; Decreasing the levels of HBsAg and HBcAg	Lai et al. (2018)
C57BL/6 mice	Intraperitoneal administration	EGCG (25 mg/kg) was given daily for 5 consecutive days	Decreasing HBV-DNA and HBV transcripts; Decreasing the levels of HBsAg, HBeAg, and HBcAg	Wang et al. (2020)
Human with genotype 4 HCV infection	Oral administration	EGCG (400 mg), sofosbuvir (400 mg), and ribavirin (1,000 mg) were given once daily for 12 or 24 weeks	More rapid rate of HCV load decline; More stable hemoglobin levels; Improving liver functions	Shiha et al. (2021)
Male C3H/HeNCrj spontaneous hepatoma mice	Oral administration	0.05% and 0.1% EGCG in drinking water were given for 65 weeks, respectively	Reducing the HCC incidence; Reducing the average number of hepatomas per mouse	Nishida et al. (1994)
Male C57BL/KsJ-db/db mice	Oral administration	0.1% EGCG in drinking water was given for 34 weeks	Preventing obesity-related liver tumorigenesis by inhibiting the IGF/IGF-1R axis, alleviating hyperinsulinemia, and reducing chronic inflammation	Shimizu et al. (2011b)
Male Sprague-Dawley rats	Intraperitoneal administration	EGCG (20 mg/kg) was given twice per week for 16 weeks	Reducing the average number of liver nodules; Elevating the survival of rats; Blocking the increase of serum AFP; Reducing the markers of oxidative stress and invasion markers	Darweish et al. (2014)
Male Wistar rats	Oral administration	EGCG-rich tea infusion was given for 20 weeks	Preventing liver tumorigenesis and reducing the HCC incidence; Ameliorating liver damage and decreasing tumor markers; Reducing the oxidative stress markers; Inhibiting inflammation in hepatocarcinogenesis process though NF-κB signaling pathway; Inducing apoptosis and inhibiting proliferation though PI3K/Akt pathway	Liao et al. (2019)
Male Sprague-Dawley rats	Intragastric administration	EGCG (25 mg/kg) was given daily	Reducing tumor volume and inhibiting tumor growth; Prolonging the survival rates of rats; Reducing the serum GGT level; Reducing CDC25A expression and upregulating p21 expression	Tang et al. (2020)
Female Swiss albino mice	Oral administration	EGCG (8 μg/kg) was given daily in different stages before or after carcinogen administration	Preventing liver carcinogenesis by reducing hepatocyte progenitor cell/stem cell population; Modulating self-renewal Wnt and hedgehog pathways; Inhibiting AFP and CD44 expressions; Determining cellular proliferation and apoptosis status	Sur et al. (2016)
Male Kunming mice	Intragastric administration	Different concentrations of tea polysaccharides (50–200 mg/kg) and tea polyphenols (15–60 mg/kg) were given once daily for 14 days	Improving antioxidant capacity and immune level synergically; Suppressing the growth and proliferation of HCC	Wang et al. (2017a)
Female BALB/c nude mice	Oral administration	0.1% or 0.5% EGCG was given for 2 weeks, respectively	Attenuating the enhanced metastatic capacity of HCC through antioxidant activity	Kang et al. (2021)
Male BALB/c nude mice with HuH7 xenograft	Oral administration	0.01% or 0.1% EGCG was given for 2 weeks for 5 weeks, respectively	Reducing tumor volume; Decreasing the expression of VEGF, VEGFR-2, ERK, Akt, and Bcl-xl	Shirakami et al. (2009)
BALB/c nude mice with HepG2 xenograft	Intragastric administration	EGCG (40 mg/kg) was given for 20 days	Suppressing tumor growth and angiogenesis; Inhibiting the expression of HIF-1α and VEGF; Suppressing the MAPK/ERK and PI3K/AKT signaling pathways	Liao et al. (2020)
Male BALB/c nude mice with HuH7 xenograft	Intraperitoneal administration, Oral administration	Methyl-EGCG (0.11 mg/kg or 1.1 mg/kg i.p., 8.3 mg/kg p.o.) was given for 21 days	Inhibiting tumor growth and angiogenesis	Hashimoto et al. (2014)
Female nude mice with Hep3B xenograft	Intraperitoneal administration	EGCG (2, 10 or 50 mg/kg) was given every other day	Inhibiting tumor growth; Inducing tumor necrosis	Chen et al. (2019)
Male BALB/c nude mice with HLE xenograft	Oral administration	0.8, 2.5 and 7.5 mg/ml EGCG in drinking water were given for 25 days, respectively	Inhibiting the growth of xenograft tumors; Inducing apoptosis and downregulating Bcl-2 and Bcl-xl	Nishikawa et al. (2006)
Male BALB/c nude mice with HCC-LM3 xenograft	Intraperitoneal administration	EGCG (10 mg/kg) was given once daily for 30 days	Suppressing tumor size; Increasing the rate of apoptosis	Li et al. (2016)
Male nude mice with Hep3B xenograft	Intragastric administration	EGCG (50 mg/kg) was given once daily for 15 days	Inhibiting tumor volume and weight; Attenuating autophagy induced by doxorubicin	Chen et al. (2014)

GGT, γ-glutamyl transpeptidase.

### Antiviral activities

In a previous study, an animal model of HBV infection was established using C57BL/6 mice in which intraperitoneal administration of EGCG for 5 consecutive days was performed. The results suggested EGCG markedly decreased HBV-DNA, HBsAg and HBeAg levels in serum, and HBV-RNA transcription as well as hepatitis B core antigen (HBcAg) expression in liver tissue (Wang et al., 2020). Similarly, in human liver chimeric mice, serological and immunohistochemical detection of liver also suggested EGCG injected through the tail vein significantly reduced HBV-DNA, HBsAg, and HBcAg levels (Lai et al., 2018). These findings further confirm EGCG can effectively suppress HBV infection, which is beneficial to block the development of HCC. Additionally, a randomized clinical trial compared the efficacy and safety of Catvira, a new formulation consisting of EGCG, sofosbuvir and ribavirin, with sofosbuvir and ribavirin tablets in patients with HCV genotype 4 (Shiha et al., 2021). The results demonstrated the patients treated with Catvira underwent a more rapid decline in viral load, with stable hemoglobin levels. In contrast, the patients treated with sofosbuvir and ribavirin suffered from a significant reduction in hemoglobin levels. This suggests EGCG not only inhibits HCV infection, but may also resist hemolysis from ribavirin treatment. However, this clinical trial reported only the results of pilot study with a small sample size, and did not include patients with pre-existing cirrhosis, HCC, and other HCV genotypes. Therefore, the effectiveness of EGCG combined with antiviral drugs remains to be further clinical trials.

### Preventive roles in tumorigenesis

Although EGCG acts a certain chemoprophylactic roles in the etiology of HCC *in vitro* and *in vivo*, the roles of EGCG in the actual HCC incidence and the corresponding molecular mechanism still need to be further elaborated. Accumulating evidence confirm EGCG can inhibit the tumorigenesis and development of HCC. A team from Japan was the first to investigate the inhibitory roles of EGCG in spontaneous hepatoma models (Nishida et al., 1994). In that study, after treating 72 mice with different concentrations of EGCG in drinking water, researchers observed EGCG minimized the HCC incidence from 83.3% to 52.2%. Later on, another study from Japan revealed EGCG attenuates obesity-related hepatocarcinogenesis though suppressing the IGF/IGF-1R axis, alleviating hyperinsulinemia, and reducing chronic inflammation (Shimizu et al., 2011b). Darweish et al. found EGCG injected intraperitoneally markedly decreased the average number of liver nodules in thioacetamide-induced rat models of HCC by 70% and elevated the survival of animals from 30% to 80% (Darweish et al., 2014). Also, previous evidence suggested ingestion of EGCG-rich tea infusion markedly diminished the size and number of hepatic neoplastic nodules induced by diethylnitrosamine, and reduced the HCC incidence from 87.50% to a staggering 8.33% (Liao et al., 2019). Further analysis indicated EGCG could regulate the hepatocarcinogenesis process though suppressing the inflammatory response caused by NF-κB and corresponding downstream signals. Simultaneously, Tang et al. uncovered EGCG prevent HCC in diethylnitrosamine-induced rat models through upregulating p21 expression and downregulating cell division cycle 25A (CDC25A) expression (Tang et al., 2020). In addition, Sur et al. deciphered EGCG could restrain the tumorigenesis of HCC through shrinking the proportion of hepatocyte progenitor cells or CSCs, and modulating Wnt and hedgehog signaling pathways, in which the downregulated β-catenin, cyclinD1, c-Myc and EGFR but upregulated E-cadherin were detected (Sur et al., 2016). The forementioned results indicates that EGCG has an obvious chemoprophylaxis effect on the tumorigenesis of HCC.

### Antioxidative activities

Unlike ordinary tea drinks, EGCG-rich green tea infusion significantly increases the antioxidant capacity in the diethylnitrosamine-induced HCC models through elevating the activity of superoxide dismutase, glutathione peroxidase, and hepatic catalase in serum, decreasing the content of malondialdehyde in liver, and reducing ROS production caused by DNA damage (Liao et al., 2019). Darweish et al. supports the above conclusion that EGCG attenuates the oxidative stress of HCC and protect liver function *in vivo* (Darweish et al., 2014). Also, in a study conducted on oolong tea extracts, the combined application of polysaccharide and polyphenols (52.17% content of EGCG) not only synergically improves the antioxidant capacity and immune level of mice, but inhibits the growth and proliferation of hepatoma cells (Wang J. et al., 2017). Subsequently, Kang et al. observed EGCG could effectively inhibit the lung metastasis of hepatoma cells after injecting luciferase-labelled hepatoma cells with overexpressed miR483-3p (a miRNA to facilitate the metastasis of HCC by inducing oxidative stress) into tail veins of mice (Kang et al., 2021). Taken together, EGCG can protect the liver from oxidative stress damage, and may even attenuate HCC progression through antioxidant effects.

### Antiangiogenic effects

Consistent with experimental results *in vitro*, EGCG significantly suppresses the growth of Huh7 xenografts in nude mice by reducing VEGF expression and inhibiting the activation of VEGFR-2 and associated downstream signaling molecules, such as Akt and ERK (Shirakami et al., 2009). Interestingly, Liao et al. (2020) directly observed EGCG and its derivative Y6 can significantly hinder the angiogenesis of chorioallantoic membrane in chicken embryos, and also clearly revealed them can significantly inhibit intratumoral angiogenesis in HepG2 xenograft models. As expected, this inhibitory effects in xenografts were found to be relevant to the inhibition of PI3K/Akt and MAPK/ERK signaling pathways and the downregulation of HIF-1α and VEGF subsequently. Similarly, Hashimoto et al. used CD31 to specifically label the Huh7 xenograft models and found that EGCG’s methylated derivatives could also significantly inhibit angiogenesis and growth of xenograft tumors (Hashimoto et al., 2014). In summary, these findings suggest EGCG has high antitumor activity *in vivo* by inhibiting the angiogenesis of HCC.

### Regulatory roles in apoptosis, autophagy, and necrosis

In addition to chemoprophylactic, antioxidant, and antiangiogenic effects have been proven *in vivo*, EGCG can also regulate the apoptosis, autophagy, and necrosis to obstruct the growth of HCC. Evidence showed abundant apoptotic cells and downregulated apoptosis suppressors, including Bcl-2 and Bcl-xl, were detected in the xenograft sections after feeding nude mice with EGCG (Nishikawa et al., 2006). Since NF-κB partially modulates the transcription of these apoptosis suppressors, researchers considered EGCG might trigger apoptosis process by inhibiting NF-κB signal. Likewise, green tea extract rich in EGCG significantly increases the expression levels of p53, caspase-3 and Bax, while decrease the expression levels of proliferating cell nuclear antigen (PCNA) in rat liver (Liao et al., 2019). Predictably, such proapoptotic effect of EGCG was further traced to the PI3K/Akt pathway. Furthermore, one research conducted on combination drugs reported that, in the HCC-LM3 xenograft models, EGCG combined with sorafenib prominently increased the percentage of apoptosis compared to monotherapy (Li et al., 2016). Another study involving drug therapy showed EGCG combined with doxorubicin increased apoptotic cells by about 50% and prominently attenuated the autophagy levels compared with doxorubicin alone in Hep3B xenograft models of nude mice (Chen et al., 2014). Moreover, *in vivo* experiments also found EGCG not only suppressed the growth of Hep3B xenograft in a dose-dependent manner, but markedly induced the necrosis of a large number of tumor cells (Chen et al., 2019). In conclusion, EGCG affects tumor growth and drug susceptibility by regulating apoptosis, autophagy, and necrosis procedures.

## Target prediction of EGCG

To further elucidate the molecular mechanism of EGCG in HCC, we supplemented the additional potential targets of EGCG through the Swiss Target Prediction (http://www.swisstargetprediction.ch/) platform, which can predict the most likely macromolecular targets for a small molecule drug (Daina et al., 2019). The results showed more than ten proteins had high probability as EGCG targets (Table 3). For instance, DNA methyltransferase (DNMT) is highly suspected as a molecular target of EGCG among them, which can transfer methyl groups from *S*-adenosine methionine to the cytosine bases of CpG dinucleotides. This epigenetic alteration results in the dysregulated expression of oncogenes and tumor suppressor genes, which is the pivotal biological processes of hepatic tumorigenesis and metastasis (Nagaraju et al., 2022). Related evidence has demonstrated EGCG induces demethylation of abnormally hypermethylated tumor suppressor genes, which may be attributed to its inhibitory roles in DNMT activity as a direct competitive inhibitor of DNMT (Moreno et al., 2016). In addition, some researchers have argued EGCG induces the downregulation and degradation of DNMT by inhibiting the association of DNMT with Ubiquitin-like with PHD and RING finger domains 1 (UHRF1), a ligase of E3 ubiquitin (Qadir Nanakali et al., 2022). These findings suggest EGCG may reduce the development of tumor by correcting the dysregulated epigenetic mechanisms. However, the effect of EGCG on epigenetic modification of HCC remains to be verified by subsequent experiments. Briefly, these potential targets may provide insights for the investigation and therapy of EGCG in HCC.

**TABLE 3:** The prediction of potential targets for EGCG.

Target	Common name	Target class	Probability^*^
DNA (cytosine-5)-methyltransferase 1	DNMT1	Writer	100.00%
Microtubule-associated protein tau	MAPT	Unclassified protein	100.00%
Dual-specificity tyrosine-phosphorylation regulated kinase 1A	DYRK1A	Kinase	100.00%
Beta amyloid A4 protein	APP	Membrane receptor	100.00%
MAP kinase p38 alpha	MAPK14	Kinase	100.00%
Telomerase reverse transcriptase	TERT	Enzyme	100.00%
Matrix metalloproteinase 2	MMP2	Protease	100.00%
6-Phosphogluconate dehydrogenase	PGD	Enzyme	100.00%
Hepatocyte growth factor receptor	MET	Kinase	100.00%
Matrix metalloproteinase 14	MMP14	Protease	100.00%
P-glycoprotein 1	ABCB1	Primary active transporter	100.00%
Beta-secretase 1	BACE1	Protease	100.00%
Apoptosis regulator Bcl-2	BCL2	Other ion channels	100.00%
Signal transducer and activator of transcription 1-alpha/beta	STAT1	Transcription factor	100.00%
Squalene monooxygenase (by homology)	SQLE	Enzyme	100.00%
HERG	KCNH2	Voltage-gated ion channel	78.78%
CMP-N-acetylneuraminate-beta-1,4-galactoside alpha-2,3-sialyltransferase	ST3GAL3	Transferase	56.99%
Alpha-(1,3)-fucosyltransferase 7	FUT7	Transferase	56.99%
Fucosyltransferase 4	FUT4	Enzyme	56.99%
GABA-A receptor; alpha-1/beta-2/gamma-2	GABRA1 GABRB2 GABRG2	Ligand-gated ion channel	10.90%

*Probability for EGCG, assumed as bioactive to have this protein as target.

## Mechanisms of EGCG in the regulation of target molecules

As mentioned above, we summarized the multiple molecular targets and signaling pathways by which EGCG plays the preventive and antitumor roles in HCC. However, how EGCG simultaneously regulates these molecular targets or signaling pathways in HCC remains to be further discussed. Computational docking analysis has demonstrated the mechanism of physical interactions between EGCG and its molecular targets involving MAPK, PI3K, Akt, NF-κB, STAT-3, RTKs, and MMPs. Specifically, Wang et al. (2019) identified extensive hydrogen bonds were formed between the hydroxyl groups of EGCG and critical residues of many vital tumor-related molecules, including MAPK and Akt, and some enzymatic activities were inhibited by EGCG *in vitro*. Van Aller et al. (2011) found the ATP binding pocket of PI3K accommodated the 3, 4, 5-trihydroxybenzoate portion of EGCG, which implies EGCG may be an ATP competitive inhibitor of PI3K. Ample evidence indicated the inhibitory effect of EGCG on NF-κB-mediated transcriptional activation was attributable to the formation of a covalent bond between EGCG and residue of NF-κB p65 subunit (Lakshmi et al., 2020; Suhail et al., 2022). Simultaneously, STAT-3 binding assay confirmed EGCG interacted with Arg-609, a critical residue in the STAT-3 SH2 domain that contributes to the binding of STAT-3 and phosphorylated peptides (Wang et al., 2013). Hsu et al. (2015) revealed that in addition to the hydroxyl groups of EGCG binding to residues F795 and E804 in EGFR via hydrogen bonds, the gallate moiety of EGCG interacted with specific anchor residues G796, C797, and D800 through van der Waals forces. Likewise, Singh and Bast also fully illustrated lipophilic, hydrophobic, electrostatic hydrogen bond, π-π stacking, and π-cationic interactions were key contributors in protein-ligand interactions between EGCG and other RTKs, including IGF-1R and VEGFR (Singh and Bast, 2015). In addition, evidence suggested EGCG interacted with several amino acid residues in MMP-9 via hydrogen bond, π-π stacking, π-cationic, and π-σ chemical interactions, and galloyl group of EGCG seem to play an important role in the inhibition of MMP-9 (Sarkar et al., 2016; Nakano et al., 2018).

On the other hand, EGCG’s regulatory effect on epigenetic events may be another underlying mechanism. DNA methylation and histone deacetylation act pivotal roles in shutting down gene expression. The former blocks the binding between transcription factors and promoters, while the latter results in a compact and inaccessible chromatin structure (Hogg et al., 2020). Scientific studies found EGCG interacted with DNMT by hydrogen bond to regulate DNA methylation, thereby reactivating a range of methylation-silenced cancer suppressor genes (Kedhari Sundaram et al., 2020; Li et al., 2022). Studies also revealed EGCG modulated histone acetylation status through docking to histone deacetylases (HDAC) and competitively inhibiting enzymatic activity (Khan et al., 2015; Negri et al., 2018). Inhibiting HDAC will lead to upregulation of histone acetylation on p21 and Bax promoters and hyperacetylation of histone H3 (Li et al., 2022). Meanwhile, inhibiting HDAC will upregulate phosphatase and tensin homolog (PTEN), resulting in Akt inactivation and PI3K/Akt pathway inhibition in HCC (Liu et al., 2021). However, the further mechanism about how EGCG regulates multiple target molecules simultaneously remains to be investigated in the future.

## The potential of EGCG in HCC therapy

Currently, there are a variety of therapeutic strategies for HCC, mainly including surgical resection, liver transplantation, percutaneous ablation, transarterial chemoembolization, radiotherapy, chemotherapy, targeted therapy, and immunotherapy (Llovet et al., 2021; Vogel et al., 2022). Although remarkable progress has been made in the systematic immunotherapy of HCC in recent years, the long-term survival of HCC patients remains unsatisfactory and the treatment of HCC still faces great challenges. Therefore, it is vital to explore new anti-cancer drugs and combined therapeutic strategies. Based on these premises, we summarize the therapeutic roles of EGCG reported in the literature.

### Chemotherapy

Chemotherapy resistance is the most intractable problem in cancer therapy, which is strong relevant to the evolution of CSCs towards drug resistance, eventually causing tumor recurrence and metastasis (Barbato et al., 2019). A study involving CSCs of hepatoma revealed EGCG attenuated the expression of ATP-binding cassette (ABC) transporters, which participate in the multidrug resistance by transporting chemotherapy drugs out of the tumor cells (Wubetu et al., 2016). This means the combination of EGCG with chemotherapeutic agents seems warranted.

5-Fluorouracil, a traditional chemotherapy agent, is still routinely used for advanced HCC. Unfortunately, non-specific cytotoxicity and chemotherapy resistance due to long-term application limit its clinical efficacy (Xu et al., 2020). Accumulating studies have found cyclooxygenase-2 (COX-2), an enzyme that catalyzes prostaglandin synthesis, is highly expressed in multiple cancers, and is involved in tumorigenicity and chemotherapy resistance, which may be related to 5-fluorouracil resistance in HCC (Zhang S. et al., 2019; Hashemi Goradel et al., 2019; Xu et al., 2020). Coincidentally, EGCG enhances the inhibitory roles of 5-fluorouracil in hepatoma growth by eliminating 5-fluorouracil-induced COX-2 overexpression and PGE2 secretion (Yang et al., 2012). This suggests that combinations of EGCG and 5-fluorouracil provide an effective clinical strategy for HCC therapy.

Daunorubicin is an anthracycline chemotherapy agent. The study of Huang et al. demonstrated EGCG overcame the chemotherapy resistance of hepatoma cells to daunorubicin and reduce the cardiotoxicity of daunorubicin through directly targeting carbonyl reductase 1 (CBR1) to inhibit the conversion of daunorubicin to daunorubicinol (Huang et al., 2010). Similarly, Y6, an ethylated derivative of EGCG, was also revealed to significantly inhibit the CBR1 expression in hepatoma cells, thus enhancing the efficacy of daunorubicin against HCC (Zhou et al., 2020).

As for doxorubicin, another anthracycline, EGCG can enhance its antitumor ability on hepatoma cells, and re-sensitize hepatoma cells resistant to it through reversing the multiple resistance mechanisms, such as suppressing multidrug resistance 1 (MDR1) expression, enhancing intracellular doxorubicin accumulation, and decreasing P-glycoprotein efflux pump activity of ABC transporter family members (Liang et al., 2010). Meanwhile, Satonaka et al. (2017) confirmed EGCG reversed the above doxorubicin resistance in hepatoma cells by synergistically inhibiting MAPK/ERK and PI3K/Akt signaling pathways. Wen et al., 2019 also confirmed EGCG derivative Y6 reversed doxorubicin resistance in hepatoma cells through competitively inhibiting efflux pump activity of ABC transporter. Additionally, Chen et al., 2014 found EGCG could kill hepatoma cells synergically with doxorubicin through inhibiting doxorubicin-induced autophagy. In conclusion, EGCG enhances the efficacy of chemotherapy through multiple mechanisms, which provides the important strategies for combination therapy of HCC.

### Radiotherapy

Besides improving the efficacy of chemotherapy agents, EGCG is also involved in the regulation of radiotherapy for HCC. It has been confirmed that hepatic stellate cells, as one of the most important components of the TME, induces HCC angiogenesis, inhibits antitumor immune response, and accelerates HCC progression (Ruan et al., 2020). Increasing evidence suggests radiotherapy activates hepatic stellate cells to reshape the TME through multiple molecular mechanisms, such as secreting cytokines, and driving hepatic fibrosis, ultimately resulting in enhanced invasion and metastasis of residual hepatoma cells (Barry et al., 2020; Ruan et al., 2020; Ezhilarasan and Najimi, 2023). This may be the reason why radiotherapy for HCC often fails. Therefore, targeting hepatic stellate cells is a novel strategy for the therapy of HCC. EGCG can suppress the activation of hepatic stellate cells though binding to laminin receptor (67 LR) to block activated toll-like receptor 4 (TLR4) signal transduction (Shen et al., 2022). Once TLR4 signal is blocked, it will be conducive to the inhibition of NF-κB and various inflammatory factors, so as to suppresses the hepatic tumorigenesis and progression. These findings suggest EGCG can be applied as an adjunct to HCC radiotherapy to reduce the invasion and metastasis abilities of residual hepatoma cells after radiotherapy.

### Targeted therapy

Sorafenib, the first multi-kinase inhibitor used in the systematic targeted therapy of HCC, which simultaneously inhibits tumor cell proliferation and angiogenesis, is still used as the first-line treatment for HCC (Huang A. et al., 2020; Benson et al., 2021). Sorafenib resistance in HCC has been confirmed to be closely relevant to the process of aerobic glycolysis (Feng et al., 2020b). Sorafenib combined with glycolysis inhibitors can markedly retarded the growth of hepatoma cells resistant to sorafenib (Xia et al., 2020). Hepatoma cells are re-sensitized to sorafenib through blocking the expression of glycolytic enzymes, including PFK and pyruvate kinase M2 (PKM2) (Li et al., 2017; Feng et al., 2020a). As expected, EGCG can increase the inhibitory effects of sorafenib on aerobic glycolytic hepatoma cells and xenograft models of mice through directly inhibiting PFK activity (Li et al., 2016). Furthermore, an interesting study demonstrated EGCG combined with low doses of sorafenib did not differ in inhibiting angiogenesis of HCC rats compared with standard doses of sorafenib, which was beneficial in preventing resistance and reducing toxicity of sorafenib (Irawan et al., 2022). Nevertheless, the application of EGCG as an adjuvant for targeted therapy of HCC remains to be further investigated clinically.

### Immunotherapy

In the systematic therapy of HCC, the therapeutic options of some patients are limited due to the resistance to traditional chemotherapy drugs. As a new treatment method, immunotherapy overturns the traditional protocols of HCC therapy, and has achieved encouraging outcomes in terms of safety and efficacy (Kole et al., 2020). So far, several ICIs have been approved for the therapy of HCC, such as atezolizumab, camrelizumab, sintilimab and tislelizumab. Unfortunately, there have been no reports of EGCG affecting the efficacy of ICIs in HCC. Even the effects of EGCG on the TME of HCC remains unclear. But we are hopeful that future research will be carried out.

## Challenge of EGCG in the therapy of HCC

Although EGCG can enhance the efficacy of chemotherapy, radiotherapy, and targeted therapy, like other natural compounds, EGCG also has its own relative limitations, such as unstable chemical structure, low oral bioavailability, and potential hepatotoxicity at high doses, which may limit its clinical application in HCC. Encouragingly, these slight deficiencies of EGCG can be improved by chemical modification or nanosystem.

### Instability

The stability of EGCG is influenced by many physicochemical factors, such as temperature, pH, oxygen concentration, and ion concentration. Among them, temperature and pH are the main reasons, since they promote the epimerization and autoxidation of EGCG (Mehmood et al., 2022). Specifically, the autooxidation mainly occurs at temperatures below 50°C, producing ROS, while the epimerization of EGCG mainly occurs at higher temperatures, producing GCG. In addition to temperature-induced instability, EGCG is highly unstable at alkaline pH, especially when pH exceeds 8, whereas that is highly stable at the acidic pH ranging from 2.0 to 5.5 (Krupkova et al., 2016b). Considering the temperature and pH factors synthetically, the degradation rate of EGCG is lowest at pH 3 and 25°C, but that is highest at pH 8 and 135°C, which implies EGCG is less stable at high temperature and alkaline pH (Xu et al., 2019). Thus, in addition to improving green tea production processes, the instability of EGCG provides guidance for future EGCG extraction, storage, and solvent selection. Notably, although EGCG remains relatively stable in gastric acid, it is less stable in the alkaline environment of the duodenum, resulting in the possibility of low bioavailability in oral administration. We hope this limitation will be further ameliorated through certain chemical modification of EGCG.

### Bioavailability

Due to the hydrophilicity and lack of active transporters of EGCG, EGCG enters intestinal epithelial cells mainly through passive diffusion (Dai et al., 2020; Alam et al., 2022). There are widely distributed efflux transporters on the surface of intestinal epithelial cells, such as P-glycoprotein, multidrug resistance-associated proteins (MRP), which may excrete intracellular EGCG into the intestinal lumen, thus reducing the absorption of EGCG (Krupkova et al., 2016a; Rashidinejad et al., 2021; Mehmood et al., 2022). In the liver, MRP on the surface of hepatocytes also mediates the excretion of polyphenols into bile (Alam et al., 2022). Besides, the metabolism of intestinal microbial flora also affects the absorption of EGCG (Gan et al., 2018). These findings imply that, in addition to the forementioned instability of EGCG in alkaline intestinal fluids, passive absorption, active excretion, and microbial decomposition may limit its oral bioavailability. According to the previous investigation of Nakagawa et al., the maximum plasma concentrations of EGCG in fasting rats and humans were 1047 ng/ml and 156 ng/ml, respectively, after treating them with 56 mg and 97 mg of EGCG orally, which further confirmed the relatively poor bioavailability of EGCG (Nakagawa and Miyazawa, 1997). Therefore, the development of lipophilic EGCG and nano-delivery system will be of substantial significance for the clinical application of EGCG in HCC.

### Hepatotoxicity

Although the time-honored tea consumption has demonstrated the safety of green tea and many green tea extracts are commercially available as dietary supplements, a growing number of researchers are concerned about their hepatotoxicity (James et al., 2018). Previously, a host of researchers believed EGCG, as a safe and non-toxic natural compound, has an inhibitory role in tumor cells and a protective effect on healthy cells (Singh et al., 2011; Niedzwiecki et al., 2016; Shiha et al., 2021). Nevertheless, subsequent animal experiments unveiled the risk of hepatotoxicity at high dosages of EGCG. Evidence suggested the intragastric administration of a 750 mg/kg once daily for 3 days caused an 80-fold increase in plasma alanine aminotransferase (ALT) levels and a 59% decrease in hepatic reduced glutathione levels of mice (James et al., 2015). In a clinical study conducted on the safety of EGCG, Siblini et al. reported that 800 mg of EGCG daily was a safe dose for reproductive-aged women, with no drug-induced liver injury in all subjects (Siblini et al., 2023). Paradoxically, in postmenopausal women, Dostal et al. reported 6.7% of them experienced elevated ALT and 1.3% of them suffered from serious adverse events when taking green tea extract containing 843 mg of EGCG daily (Dostal et al., 2015). Although the two clinical trials used similar doses of EGCG, even from the same country, the reasons for the contrary results of hepatotoxicity may be related to age and race of the patients. Overall, even though the hepatotoxicity of EGCG remains controversial, we believe there is sufficient evidence to doubt the hepatotoxic effects at high doses of EGCG. Several researchers suggested 338 mg EGCG ingested as solid doses of tea preparations daily was a safe level for adults based on toxicology and human safety data, while 704 mg EGCG ingested as beverages of tea preparations daily might be an observed safe level based on human adverse event data (Hu et al., 2018). Unfortunately, the safe dose range of EGCG for the patients with HCC remains to be determined based on clinical practice.

## Application of EGCG nanosystems in HCC

As an emerging tumor therapy, nanosystems have been widely investigated and applied in recent years (Li et al., 2021). Many natural compounds are coated and delivered by nanosystems to regulate drug release, increase biofilm permeability, alter distribution in the body, which helps to improve the bioavailability and reduce the dosage-induced hepatotoxicity (Gan et al., 2018; Aiello et al., 2021). Therefore, the application of nanosystems is expected to address the challenges of EGCG in the therapy of HCC.

Several EGCG nanosystems have been found to inhibit HCC. Tang et al. designed the drug-delivery nanoparticles loaded with EGCG-functionalized chitin and honokiol, which significantly reduced mitochondrial membrane potential, arrested cell cycle, and inhibited the HCC proliferation (Tang et al., 2018). Mostafa et al. found EGCG-capped gold nanoparticles had better antitumor effects on HCC than EGCG alone (Mostafa et al., 2020). After treatment with this gold nanoparticles, the expressions of caspase-3 and tumor suppressant factors were upregulated, while the expression of c-Myc proteins were decreased in hepatoma cells. Analogously, Gao et al. confirmed EGCG-loaded gold nanocages with photothermal effect significantly upregulated the levels of apoptosis-related proteins in HepG2 cells, including caspase-9, caspase-3 and Bax, whereas inhibited the levels of Bcl-2 (Gao et al., 2022). Moreover, Zhang et al. utilized EGCG and ursolic acid to construct a new “core shell” co-assembly carrier-free nanosystem, which could synergize immunotherapy via activating innate immunity and acquired immunity (Zhang et al., 2021). In conclusion, the development of EGCG nanosystems provides new insights and interventions for the therapy of HCC.

Although the technology of drug-delivery nanosystems in HCC is becoming more sophisticated, the suitable encapsulation methods for EGCG remain limited. In addition, there are still few studies on the safety of EGCG-delivery nanosystems, and no comparison of the efficacy between different nanosystems loaded EGCG in the therapy of HCC. Thus, there is still a host of work and challenges to be done and overcome before EGCG-delivery nanosystems are available clinically.

## Clinical studies of EGCG in HCC

Ample preclinical studies have confirmed that EGCG has prominent antitumor effects and comprehensive therapeutic advantages from multiple perspectives. To date, the corresponding clinical trials about the antitumor roles of EGCG have also been performed in several tumors according to ClinicalTrials.gov (www.clinicaltrials.gov) database (Table 4). However, there is still short of relevant intervention trials to unveil the chemoprophylaxis and clinical efficacy of EGCG in HCC. An early clinical trial with limited participants only explored the safety and tolerability of EGCG in patients with hepatic cirrhosis, but did not examine whether EGCG alleviated the pathological changes or clinical symptoms of cirrhosis, nor did it follow up the incidence of HCC (Halegoua-De Marzio et al., 2012). In recent years, a promising clinical trial (NCT03278925) has been registered in the ClinicalTrials.gov database (www.clinicaltrials.gov) to investigate how well EGCG works in prevents HCC in patients with cirrhosis, but no results have been posted or published yet. The results of clinical trials from other oncology fields may provide some references for the therapeutic effect of EGCG in HCC. For example, a clinical randomized controlled trial has confirmed that green tea extract with EGCG as the main active ingredient has the expected safety, which can prevent the colorectal neoplasia to some extent during 3-year follow-up period (Seufferlein et al., 2022). However, the development of colorectal tumors is a relatively long process, and further extension of follow-up period may result in more than expected outcomes. Another phase II preoperative randomized controlled trial involving patients with bladder cancer showed EGCG-rich green tea extract significantly reduced biological markers of bladder cancer tissue, including PCNA and clusterin, which are associated with tumor proliferation, invasion, and migration (Gee et al., 2017). In view of this, we speculate EGCG is promising to show outstanding efficacy in future clinical trials for HCC.

**TABLE 4:** The clinical trials involving the antitumor roles of EGCG.

Condition/Disease	Start year	Study phase	Sample size	Intervention	Main outcome measures	Study identifier
Breast cancer	2023	Early Phase 1	200	300 mg EGCG, 500 mg quercetin, 50 mg zinc sulfate, and 850 mg metformin daily during chemotherapy courses	3- and 10-year disease-free survival, 10-year breast cancer-specific survival, total patient chair time of drug administration, and treatment-related adverse events	NCT05680662
Breast cancer	2009	Phase 2	1,075	800 mg EGCG daily for 12 months	Mammographic density, and circulating concentrations of reproductive hormones and IGF axis proteins	NCT00917735
Prostate cancer	2021	Phase 2	360	EGCG-enriched green tea catechins twice a day for 6 months	Expression of cellular proliferation markers, apoptosis ratio, PSA, EGCG concentrations, incidence of adverse events, and changes in quality of life	NCT04597359
Prostate cancer	2020	Phase 2	135	405 mg EGCG twice a day for 24 months	Progression rate, occurrence of adverse events, PSA, and proportion of men with no cancer in the post-intervention biopsy	NCT04300855
High-grade prostatic intraepithelial neoplasia	2007	Phase 2	97	200 mg EGCG twice a day for 12 months	Incidence of progression to prostate cancer, PSA, change in scores of lower urinary tract symptom, and treatment-related adverse events	NCT00596011
Bladder cancer	2008	Phase 2	31	4 or 6 capsules of EGCG-enriched green tea catechin daily for 14–28 days	EGCG levels in tumor and normal tissue, and IGF-1 levels in serum	NCT00666562
Uterine leiomyoma	2023	Phase 3	200	1,650 mg green tea extract (45% EGCG) daily for 6 months	Tumor volume, fibroid symptom severity score, conception rate, and cumulative live birth rate	NCT05364008
Uterine leiomyoma	2022	Phase 3	60	300 mg EGCG, 50 mg D-chiro-inositol, 10 mg Vitamin B6, and 50 μg Vitamin D daily for 3 months	Diameter and volume of tumor, percentage of need for surgery, phosphorylation of VEGFR, and expression of cellular proliferation markers	NCT05448365
Uterine leiomyoma	2022	No	108	150 mg EGCG, 25 mg D-chiro-inositol, 5 mg Vitamin B6, and 25 μg Vitamin D twice a day for 3 months	Volume of the larger fibroid, changes in quality of life, and treatment-related side effects	NCT05409872
Ovarian cancer	2008	Phase 2	16	500 ml EGCG-enriched green tea drink daily until recurrence or for 18 months	Time to relapse, and toxicity	NCT00721890
Colon cancer	2017	Early Phase 1	50	450 mg EGCG twice a day within 4–12 weeks of surgery	Change in methylation from baseline compared to the control	NCT02891538
Colorectal adenomas	2011	Phase 2	1,001	150 mg EGCG twice a day for 3 years	Incidence of colorectal cancer, incidence of metachronous colorectal adenomas, and number and size of colorectal adenomas	NCT01360320

PSA, prostate-specific antigen.

Luckily, numerous observational studies have confirmed that tea consumption is notably relevant to a reduced risk of HCC (Kim et al., 2020). A comprehensive analysis of cohort and case-control studies demonstrated the summary relative risk of HCC among the highest tea drinkers (≥5 cups daily) decreased to 0.62 (95% confidence interval: 0.49–0.79) compared with non-tea drinkers (Ni et al., 2017). Similarly, in Asian, the summary relative risk for the populations with the highest green tea consumption was 0.88 (95% confidence interval: 0.81–0.97) (Huang Y. Q. et al., 2016). To a certain extent, these studies provide evidence for the role of EGCG in the prevention of HCC. Nevertheless, other bioactive ingredients contained in green tea, such as caffeine, polysaccharides, and vitamins, may have a confounding effect on the hepatic tumorigenesis, even if they are not present in the largest amounts. Hence, further specific high-level prospective randomized controlled trials need to be conducted to further confirm the efficacy of EGCG in the future.

## Future perspectives

Currently, there is a lack of high-quality clinical trials of EGCG in HCC. Even though EGCG has shown significant antitumor evidence in HCC in animal models, we remain ignorant about whether EGCG can also effectively intervene in the development and improve the prognosis of HCC. Therefore, large sample size and multi-center clinical trials are urgently needed to confirm the safety and efficacy of EGCG in HCC, which may become the future focus of research on natural polyphenol compounds in the field of HCC therapy.

Considering the hepatotoxicity caused by high-dosage EGCG, when investigating the clinical efficacy of EGCG in HCC in the future, we should closely monitor the changes in liver function of patients, especially those who have suffered from cirrhosis or partial hepatectomy. Also, we should focus on finding the optimal dose that can inhibit hepatoma cells without damaging the function of normal hepatocytes. Simultaneously, greater efforts should be made to design the EGCG derivatives and EGCG-delivery systems to help control drug release and increase the stability, bioavailability, and bioactivity in response to the challenges of future clinical applications. Remarkably, preclinical studies have demonstrated that melatonin and EGCG are ideal partners in the therapy of HCC. Melatonin has the dual roles in attenuating the hepatotoxicity and enhancing the anti-cancer potential of EGCG (Wang et al., 2015; Zhang et al., 2019a). Therefore, the combination of EGCG and melatonin is also promising to be a novel therapeutic strategy for HCC in the future.

Although EGCG enhances sensitivity to chemotherapy, radiotherapy and targeted therapy in HCC, the roles of EGCG in HCC immunotherapy have been poorly investigated *in vitro* and *in vivo*. Even, the roles and mechanisms of EGCG in the tumor immune microenvironment of HCC remain obscure. Recent research has suggested EGCG inhibits the expression of PD-L1 and PD-L2 in melanoma by targeting the Janus kinase (JAK)/STAT signaling pathway and its downstream transcriptional regulator IRF1, causing the reactivation of the immune response of cytotoxic T lymphocytes (Ravindran Menon et al., 2021). More surprisingly, although EGCG does not work through blocking immune checkpoints, its inhibitory roles in tumor growth are comparable to ICIs. Therefore, we speculate EGCG could increase the therapeutic effects of ICIs, and might even be an alternative therapeutic strategy to block the PD-L1/PD-1 axis. Based on this premise, it is essential to explore the association between EGCG and tumor immune microenvironment of HCC in the future, so as to provide evidence for combined therapy of EGCG and ICIs.

In addition, the antitumor roles of EGCG may involve the mobilization of intracellular copper ions. It was found that with the increase of intracellular copper ion levels, DNA breakage in hepatoma cells induced by EGCG was also increased, which may be correlated with the fact that EGCG produces ROS in a pro-oxidative manner by targeting copper accumulation in hepatoma cells (Farhan et al., 2015). However, recent studies have discovered intracellular accumulation of copper can trigger cuproptosis, a novel copper-induced mode of mitochondrial cell death (Tang et al., 2022; Tsvetkov et al., 2022). Perhaps, there are a partial correlation between the antitumor effects of EGCG and cuproptosis. This still needs to be validated by a host of experimental data in the future.

## Conclusion

In summary, sufficient evidence confirms EGCG suppresses the tumorigenesis and progression of HCC by various molecular mechanisms *in vitro* and *in vivo*. Furthermore, EGCG significantly enhances the efficacy of chemotherapy, radiotherapy, and targeted therapy, which provides a novel therapeutic strategy for HCC. Nevertheless, we still need to conduct large sample size, multi-center clinical trials to investigate the safety and efficacy of EGCG in HCC therapy to further promote its clinical application in the future.

## Figures and Tables

**Table:** 

**ABC**	adenosine triphosphate (ATP)-binding cassette
**ACC**	acetyl CoA carboxylase
**AFP**	α-fetoprotein
**Akt**	protein kinase B
**ALT**	alanine aminotransferase
**AMPK**	adenosine monophosphate-activated protein kinase
**ATG5**	autophagy-related protein 5
**Bcl-2**	BCL2 apoptosis regulator
**CBR1**	carbonyl reductase 1
**CDC25A**	cell division cycle 25A
**c-FLIP**	cellular FLICE-like inhibitory protein
**COX-2**	cyclooxygenase-2
**CSCs**	cancer stem cells
**CYP2E1**	cytochrome P4502E1
**DNMT**	DNA methyltransferase
**DR4**	death receptor 4
**DR5**	death receptor 5
**EGCG**	epigallocatechin gallate
**EGFR**	epidermal growth factor receptor
**EP1**	prostaglandin E receptor
**ERK**	extracellular signal-regulated kinase
**ERα36**	estrogen receptor alpha 36
**FASN**	fatty acid synthase
**FXRα**	farnesoid X receptor alpha
**GGT**	γ-glutamyl transpeptidase
**GRP78**	adenosine triphosphate (ATP)-binding domain of 78 kDa glucose regulated protein
**GSK-3β**	glycogen synthase kinase-3β
**HBcAg**	hepatitis B core antigen
**HBeAg**	hepatitis B e antigen
**HBsAg**	hepatitis B surface antigen
**HBV**	hepatitis B virus
**HCC**	hepatocellular carcinoma
**HCV**	hepatitis C virus
**HDAC**	histone deacetylase
**HER2**	epidermal growth factor receptor type 2
**HIF-1**	hypoxia-inducible factor-1
**HMG-CoA**	3-hydroxy-3-methylglutaryl-coenzyme A
**HNF4α**	hepatocyte nuclear factor 4α
**ICIs**	immune checkpoint inhibitors
**IFN-λ1**	interferon lambda 1
**IGF**	insulin-like growth factor
**IGF-1R**	insulin-like growth factor-1 receptor
**ISGs**	IFN-stimulated genes
**JAK**	Janus kinase
**LC3**	microtubule-associated protein light chain 3
**MAPK**	mitogen-activated protein kinase
**MDR1**	multidrug resistance 1
**MMPs**	matrix metalloproteinases
**MRP**	multidrug resistance-associated protein
**mTOR**	mechanistic target of rapamycin
**Nek2**	never in mitosis A (NIMA)-related kinase 2
**NF-κB**	nuclear factor-kappaB
**NTCP**	sodium taurocholate co-transporting polypeptide
**PARP**	poly (ADP-ribose) polymerase
**PCNA**	proliferating cell nuclear antigen
**PFK**	phosphofructokinase
**PGE2**	prostaglandin E2
**PI3K**	phosphatidylinositol-3-kinase
**PKM2**	pyruvate kinase M2
**PSA**	prostate-specific antigen
**PTEN**	phosphatase and tensin homolog
**RIG-I**	retinoic acid-inducible gene I
**ROS**	reactive oxygen species
**RTKs**	receptor tyrosine kinases
**STAT3**	signal transducer and activator of transcription 3
**TLR3**	toll-like receptor 3
**TLR4**	toll-like receptor 4
**TME**	tumor microenvironment
**TRAIL**	tumor necrosis factor (TNF)-related apoptosis-inducing ligand
**UHRF1**	Ubiquitin-like with PHD and RING finger domains 1
**VEGF**	vascular endothelial growth factor
**VEGFR**	vascular endothelial growth factor receptor
**67 LR**	laminin receptor
